# Novel Biomarkers for the Diagnosis of Urinary Tract Infection—A Systematic Review

**DOI:** 10.4137/bmi.s3155

**Published:** 2009-08-05

**Authors:** Neha Nanda, Manisha Juthani-Mehta

**Affiliations:** Department of Internal Medicine, Section of Infectious Diseases, Yale University School of Medicine, New Haven, CT, USA. Email: neha.nanda@yale.edu

**Keywords:** urinary tract infection, novel biomarkers

## Abstract

Urinary tract infections (UTIs) are associated with significant morbidity. We rely on clinical presentation, urinalysis, and urine culture to diagnose UTI. To differentiate between lower UTI and pyelonephritis, we depend on the clinical presentation. In the extremes of age and in immunocompromised individuals, clinical presentation is often atypical posing a challenge to diagnosis. In the elderly, the high prevalence of asymptomatic bacteriuria is another confounder. We conducted a search of publications to find novel biomarkers to diagnose UTI and to ascertain its severity. We searched PUBMED, MEDLINE and SCOPUS databases for studies pertaining to novel biomarkers and UTI. Two reviewers independently evaluated the methodology of the studies using the STARD (Standards for Reporting of Diagnostic Accuracy) criteria. We have identified procalcitonin as a biomarker to differentiate lower UTI from pyelonephritis in the pediatric age group. Elevated serum procalcitonin levels can result in early and aggressive treatment at the time of presentation. Interleukin 6 has also shown some promise in differentiating between lower UTI and pyelonephritis but needs further validation. Lastly, given the paucity of data in certain subgroups like diabetics, kidney transplant recipients, and individuals with spinal cord injury, further studies should be conducted in these populations to improve diagnostic criteria that will inform clinical management decisions.

## Introduction

Urinary tract infection (UTI) is a common bacterial infection in all age groups. The prevalence in infants is 6.5% and 3.3% in girls and boys, respectively.^1^ Among infants, approximately 60% of febrile UTIs will result in renal scarring which increases the risk of secondary hypertension.^2^,^3^ In adults age 65 years or older, it is the second most common cause of infectious disease related hospitalizations.^4^ UTI accounts for up to 30% of all infectious complications in kidney transplant recipients. UTI appearing in the first three months of kidney transplantation is associated with pyelonephritis, bacteremia, and allograft dysfunction.^5^ In individuals with spinal cord injuries (SCI), UTI is the second most common cause of death.^6^ Catheter associated UTI is the most common nosocomial infection in US hospitals and nursing homes.^7^ Other populations predisposed to UTI include diabetics and individuals with polycystic kidney disease. Despite significant morbidity associated with UTI, making a diagnosis and establishing the severity of infection in the urinary tract remains a challenge.

Establishing the severity and extent of infection (i.e. lower UTI vs. pyelonephritis) is important to determine further management. The duration of treatment in uncomplicated UTI (i.e. involving lower urinary tract) is generally shorter compared to complicated UTI (i.e. involving renal parenchyma).^8^ If there is renal parenchymal involvement, the antibiotic of choice would be one that has excellent penetration in the renal parenchyma. Untreated pyelonephritis in young children can lead to secondary hypertension because of renal scarring.^3^ Acute pyelonephritis at the extremes of age and in immunocompromised individuals can result in sepsis, renal abscess, hydronephrosis and rarely xanthogranulomatous pyelonephritis.^9^ The typical clinical presentation of lower UTI includes frequency, urgency, and dysuria. Classic symptoms of pyelonephritis include fever, flank pain along with lower UTI symptoms. Often the absence of fever indicates lower UTI and not upper UTI.^10^ Multiple studies have shown that clinical characteristics alone are inadequate to localize the site of infection in the urinary tract.^11^–^13^ Biggi et al showed that high fever was not a reliable sign to discriminate pyelonephritis from lower UTI. Maximum temperature in the lower UTI group and pyelonephritis group was 39 ± 0.7 °C and 39.2 ± 0.6 °C, respectively (P = 0.91). The sensitivity and specificity of temperature more than 39.1 °C, to diagnose pyelonephritis, was 64% and 38% respectively.^11^ In another study by Farnsworth et al of the 29 individuals, 12 (41%) had parenchymal involvement but were afebrile.^12^

With the aforementioned limitations of clinical evaluation, biological markers (biomarkers) may have the potential to diagnose UTI and determine the severity of infection. A biomarker is defined as “a characteristic that is objectively measured and evaluated as an indicator of normal biological processes, pathogenic processes, or pharmacologic responses to a therapeutic intervention.”^14^ To differentiate between lower UTI and pyelonephritis, biomarkers like erythocyte sedimentation rate (ESR), C-reactive protein (CRP) and white blood count (WBC) have been studied in children. The sensitivity of ESR to determine renal parenchymal damage varies with the cut off. A retrospective study, using a cut off of >10 mm/h for ESR yielded a sensitivity and specificity of 100% and 8%.^13^ A prospective study using a cut off >68 mm/hr for ESR yielded a sensitivity and specificity of 48% and 50% respectively. For CRP, to diagnose pyelonephritis, the sensitivity was 64% and specificity was 68%. White blood count of more than 14,601 cells/mm^3^ has a sensitivity and specificity of 56% and 58% to diagnose pyelonephritis.^11^ Given the variable results of these biomarkers, it is challenging to use these biomarkers alone to detect renal parenchymal damage and therefore we rely on radiological studies. Further, to make a diagnosis of lower UTI, we use urinalysis and urine culture results. Given the high incidence of asymptomatic bacteriuria (ASB) and no evidence to treat ASB in the general population, differentiating between UTI and ASB is imperative. Leukocyte esterase and nitrite are biomarkers measured in urinalysis for diagnosing UTI. The absence of either of these biomarkers makes either condition extremely unlikely.^15^ However, the presence of either or both of these biomarkers does not assist in differentiating ASB from UTI. Therefore, novel biomarkers that can assist in the diagnosis and determination of severity of disease for UTI need to be identified.

## Methods

### Studies eligible for review

Studies evaluating serum and urine biomarkers for the diagnosis of UTI in humans were included. Prospective, retrospective, cohort, case control studies, or randomized controlled trials were included.

### Finding relevant studies

PUBMED, MEDLINE and SCOPUS databases were searched for “urinary tract infection”, “biological marker” and “diagnosis” and then limited to studies published in the English language from 1997 through 2008 in human subjects. After selecting and reviewing relevant articles, we identified potential novel biomarkers. We identified novel biomarkers based on the number and quality of studies conducted for a given biomarker. Then we searched all databases (PUBMED, MEDLINE and SCOPUS) using “urinary tract infection” with each potential novel biomarker, with the same limits noted above.

### Quality assessment

The STARD (Standards for Reporting of Diagnostic Accuracy) criteria were utilized to assess the validity of the studies.^16^ The studies were graded based on a checklist that included nine STARD criteria relevant to this review (see Table 1). Studies were divided into three categories based on scores. A score of 8–9 indicates good quality, 6–7 indicates fair quality, and 5 or less is categorized as poor quality. Two reviewers independently graded the studies. Disagreements were discussed until a consensus was reached.

## Results

**Figure 1:**
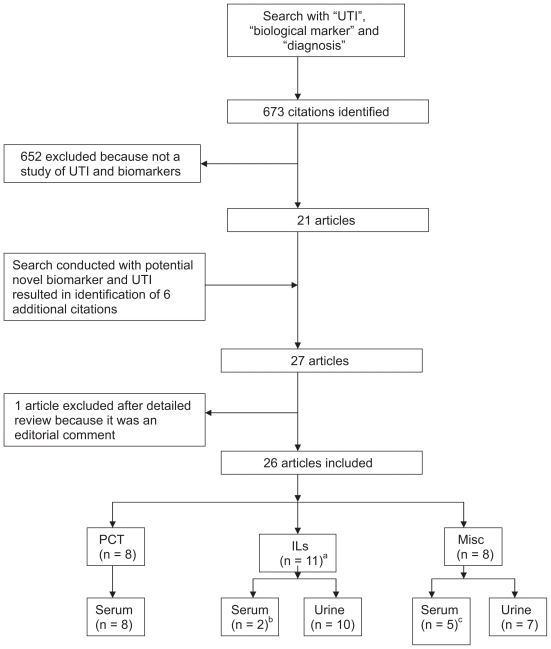
Selection of studies. ^a^One study discussing PCT and IL; ^b^One study discussing IL in both serum and urine; ^c^Four studies discussing biomarkers in both serum and urine. **Abbreviations:** UTI, urinary tract infection PCT, procalcitonin ILs, interleukins.

Six hundred seventy three citations were identified with the initial search, of which 21 articles were selected. Articles that were excluded were not relevant to our study question, i.e. did not discuss UTI and novel biomarkers. We identified procalcitonin (PCT), interleukin 6 (IL-6) and interleukin 8 (IL-8) as potential novel biomarkers for diagnosing urinary tract infection and assessing the severity of infection. With the second search (“urinary tract infection” with “PCT” or “IL-6” or “IL-8”), six additional articles were retrieved. A full text analysis of 27 articles resulted in the inclusion of 26 studies (see Fig. 1). Two studies were deemed of good quality and fourteen studies were of fair quality. The remaining studies were of poor quality. One study was not scored because of inadequate information (see Tables 2 and 3).

All studies deemed as good and fair quality are discussed in this review.

### Procalcitonin (PCT)

Procalcitonin (PCT) is a propeptide of calcitonin. It was first described by Assicot et al as a marker of bacterial invasion.^17^ In healthy humans, it is produced in the C-cells of the thyroid gland, and during a severe bacterial infection, it is produced by the monocyte-macrophage system. In humans, after the 3rd day of life, a normal plasma PCT level is less than 0.5 μg/l.

Eight articles studying serum PCT and urinary tract infection were identified. One study was of good quality, five were fair and the remaining were of poor quality.^18^ Most of these studies were conducted in the pediatric age group with an aim to differentiate between acute pyelonephritis and lower UTI. The reference standard for parenchymal damage in all of these studies was 99mTc-dimercaptosuccinic acid (DMSA) scintigraphy.

Benador et al conducted a prospective study in 80 children who were 1 month to 16 years of age. At presentation, PCT, C reactive protein (CRP) and white blood cell count (WBC) were compared in children with pyelonephritis and lower UTI.^18^ There were statistically significant differences between the two groups for all three parameters, but only PCT had a good correlation with increasing severity of scintigraphic changes noted on DMSA. In the pyelonephritis group, PCT was 5.37 μg/L (95% confidence interval [CI] 1.57 to 9.17) and 0.38 μg/L (95% CI 0 to 0.76) in the lower UTI group. Gürgoze et al conducted a prospective study in 76 children with UTI.^19^ There were 34 subjects in the acute pyelonephritis group and 42 in lower UTI group. Median PCT level obtained at presentation was significantly higher in the acute pyelonephritis group, i.e. 1.68 ng/ml (range 0.14 to 5.4) vs. 0.1 ng/ml (range 0.1 to 3.2), P < 0.001. Using a cut-off of 0.5 ng/ml, to diagnose pyelonephritis, the sensitivity and specificity of PCT was 94% and 58% respectively.

In a smaller group of children (n = 54), Grevaix et al also confirmed a higher level of PCT in the setting of parenchymal damage compared with lower UTI.^20^ In another prospective study of 64 children, Smolkin et al demonstrated that in acute pyelonephritis, the median PCT was 3.41 μg/L (range 0.36 to 12.4) while in lower UTI, the median PCT was 0.13 μg/L (range 0.02 to 2.15), P < 0.0001.^21^ In this study, using a cut-off of 0.5 μg/L, PCT had a sensitivity of 94.1% and specificity of 89.7% for detecting pyelonephritis.

Pecile et al prospectively showed that PCT can serve as a marker of severity of acute pyelonephritis in children.^22^ The study included 100 children. Mean PCT increased proportionally with the extent of renal involvement (P < 0.0001). PCT levels at admission were lower in patients with completely reversible lesions (mean 3.25 ng/ml, 95% CI 0 to 10.25) versus those with partially reversible lesions or renal scarring (mean 7.48 ng/ml, 95% CI 0 to 24.28), P = 0.04. A cutoff value of 0.8 ng/ml yielded the best diagnostic accuracy with a sensitivity of 83.3% and specificity of 93.7% to diagnose acute pyelonephritis.

Lastly, Guven et al conducted a prospective study with 33 children and were unable to show a significant correlation between parenchymal damage and PCT, CRP and WBC. 23 Subjects were enrolled based on presenting symptoms and diagnosis of acute pyelonephritis was confirmed with DMSA. In summary, PCT is a good marker to predict renal parenchymal damage in the pediatric age group. It has not been studied in other populations to make a diagnosis of UTI or to identify renal parenchymal damage as a result of infection.

### Interleukins (ILs)

Interleukins are a family of cytokines that play a key role in the regulation of the immune system. We identified 11 articles studying interleukins in the setting of UTI. Seven articles were graded as fair quality and the rest were poor quality. Interleukin 6 (IL-6) and interleukin 8 (IL-8) have been studied in adults to diagnose UTI and in children to differentiate between pyelonephritis and lower UTI.

Urinary IL-8 has been suggested as a biomarker for UTI in catheterized postoperative patients. In a study by Olsyzna et al, urinary IL-8 and IL-6 were measured prospectively.^24^ A diagnosis of UTI was made if the patient had at least one clinical symptom of UTI plus greater than 100,000 CFU/ml on urine culture. One hundred and sixty five patients were initially enrolled and 10 were diagnosed with UTI. Twenty patients, matched for duration of catheterization, served as controls. Urinary IL-8 increased on the day the urine culture became positive in the UTI group, while in the control group it was unchanged. Urinary IL-8 increased from approximately 10 to 125 ng/mmol of creatinine in the UTI group and remained constant at approximately 10 ng/mmol of creatinine in the control group. Urinary IL-6 increased in both the case and control groups.

Jantausch et al prospectively studied urinary IL-6 and IL-8 as markers for UTI in children age 0–12 years.^25^ Bacterial UTI was defined as a urine culture with greater than 100,000 CFU/ml in a midstream clean catch specimen or greater than 10,000 CFU/ml in a catheterized specimen. The control group included febrile subjects with infections other than UTI. At the time of admission, the median urinary IL-6 concentration for subjects (n = 37) with proven bacterial UTI was 397 pg/ml (range 0 to 65,790 pg/ml) vs. 0 pg/ml (range 0 to 473.8 pg/ml) for controls (n = 37), P < 0.0001. The median urinary IL-8 concentration for the bacterial UTI group (n = 32) was 5809 pg/ml (range 0 to 347,368 pg/ml) vs. 0 (range 0 to 2231 pg/ml) for the controls (n = 32), P < 0.0001.

Sheu et al compared the utility of serum and urine levels of IL-6 and IL-8 for diagnosing acute pyelonephritis.^26^ Seventy eight children aged 1–121 months with a first episode of febrile UTI were included. Twelve healthy children who were matched for age and sex served as controls. All patients with suspected UTI received antibiotics at the time of presentation. Serum and urine IL-6 and IL-8 were collected at presentation. Acute pyelonephritis was confirmed with DMSA. The initial value of both interleukins in serum and urine was significantly higher in patients with acute pyelonephritis (n = 42) compared with lower UTI (n = 36) and healthy controls (n = 12). Serum IL-6 was 67.5 ± 75.4 pg/ml (mean ± standard deviation [SD]) in the pyelonephritis group, 12.1 ± 15.0 pg/ml in the lower UTI group and 1.6 to 2.8 pg/ml in the healthy controls, P < 0.001. Urine IL-6 was 516 ± 685.9 pg/mg of creatinine (mean ± SD) in the pyelonephritis group, 46.9 ± 78.8 pg/mg in lower UTI group and was undetectable in healthy control group, P < 0.001. IL-6 in serum and urine significantly correlated with each other and with fever, CRP and leukocytes in the urine. Serum IL-8 was 29.2 ± 27.3 pg/ml (mean ± SD) in the pyelonephritis group, 7 ± 9.2 pg/ml in lower UTI group and 1.5 ± 3.6 pg/ml in the healthy controls, P < 0.001. Urine IL-8 was 3165.8 ± 4665.1 pg/mg of creatinine (mean ± SD) in the pyelonephritis group, 172 ± 350.8 pg/mg in the lower UTI group and 11.2 ± 15.1 pg/mg in the healthy control group, P < 0.001. Serum IL-8 and urinary IL-8 significantly correlated with each other but not with clinical symptoms. Of note, the standard deviation was larger than the mean value in most cases. Serum and urine IL-6 had a higher sensitivity and specificity to diagnose acute pyelonephritis compared to serum and urine IL-8.^29^ Sensitivity and specificity for serum IL-6, using a cut-off value of 22 pg/ml was 88% and 83%, respectively. For urine IL-6, using a cut-off value of 70 pg/mg of creatinine, sensitivity and specificity was 86% and 81% respectively.

Gürgoze et al showed that at the time of presentation, median serum IL-6 was higher in the pyelonephritis group compared with the lower UTI group, 59 pg/ml (range 0–357.2) vs. 10 pg/ml (range 0–64), P < 0.001.^19^ This study was conducted in children less than one year of age. To diagnose pyelonephritis, using a cutoff of 18 pg/ml for serum IL-6, yielded a sensitivity of 88% and a specificity of 74%.

Contrary to Sheu et al, Krzemień et al showed no difference in urinary IL-6 and IL-8 in subjects with acute pyelonephritis and UTI. Krzemień et al studied 33 children in the age group of 1–24 months who were admitted with the first episode of UTI. Urinary IL-6 measured as urinary IL-6/creatinine was not significantly different in the pyelonephritis and UTI groups (median 2.83 pg/mg, range 0 to 122.55 vs. 3.81 pg/mg, range 0 to 41.67).^27^ Median urinary IL-8 measured as urinary IL-8/creatinine was 45.12 pg/mg (range 0 to 3200.77) in the pyelonephritis group and 0 pg/ml (range 0 to 117.45) in the UTI group. The difference between the two groups was not significant.

Otto et al prospectively studied the serum chemokine profile of adults (age 18–85 year old) with febrile UTI.^28^ Serum IL-8 concentration was higher in patients with clinical signs of acute pyelonephritis than in the group with lower UTI symptoms. In the pyelonephritis group, median IL-8 was 1.45 ng/ml (range 0.31 to 7.70) and 0.88 ng/ml (range 0.20 to 4.10) in the lower UTI group but the difference was not significant.

IL-1 is another immunoregulatory cytokine. IL-1β is a subtype of IL-1, found in free form in biological fluids like serum, urine, and synovial fluid. IL-1β has also been studied to differentiate between acute pyelonephritis and lower UTI. In addition to investigating PCT and IL-6, Gurgoze et al determined that serum IL-1β had a sensitivity of 97% and a specificity of 59% for detecting pyelonephritis.^19^ A cut-off of 6.9 pg/ml was used to distinguish between pyelonephritis and lower UTI. In the pyelonephritis group, median IL-1β was 32.3 pg/ml (range 1.63 to 70.2) and in the lower UTI group, median IL-1β was 1.64 pg/ml (range 0 to 14.55), P < 0.001. Similarly, urinary IL-1β proved to be a helpful marker in another study comparing patients with acute pyelonephritis (n = 41), lower UTI (n = 34), and febrile controls (n = 20, patients with upper respiratory infection, lower respiratory infection, otitis media, and gastroenteritis).^29^ There was a significant difference in IL-1β levels at presentation in all three groups; acute pyelonephritis (172 ± 263.3 pg/ml, mean ± SD), lower UTI (20.4 ± 41.2 pg/ml) and febrile controls (4.6 ± 14 pg/ml).

IL-6 and IL-8 have been studied in several settings including diagnosing UTI in adults in the postoperative setting, in diagnosing bacterial UTI in children, and in diagnosing pyelonephritis in children. Serum IL-6 is a possible novel biomarker to diagnose acute pyelonephritis in children but further studies are warranted. Currently, there are discrepant results when using urinary IL-6, serum and urinary IL-8 to diagnose acute pyelonephritis in children and adults.

### Miscellaneous

Besides PCT and IL, other novel biomarkers have been studied to diagnose UTI. We identified eight articles discussing these biomarkers. Among these, the article regarding polymorphonuclear elastase was rated as a good quality article. Studies discussing urinary lactoferrin and urinary secretory IgA were fair quality based on our scores.

Elastase is a protease stored in neutrophils. It is present in plasma and urine in a formed complex (elastase-a_1_-antitrypsin complex, E-a_1_-Pi). Fretzayas et al investigated the utility of E-a_1_-Pi for detecting pyelonephritis in children with UTI.^30^ Eighty three children below the age of 14 were enrolled. The first episode of symptomatic UTI with a single organism (≥10^5^ CFU/ml) with or without fever was the inclusion criteria. DMSA was used to confirm the diagnosis of pyelonephritis. Plasma E-a_1_-Pi levels were significantly elevated in patients with pyelonephritis (n = 30) compared with patients without parenchymal involvement (n = 53), 103.5 ± 9.3 μg/ml vs. 54.1 ± 6.5 μg/ml (mean ± standard error) respectively, P < 0.01. Urinary E-a_1_-Pi showed no significant difference between the two groups.

Polymorphonuclear leukocytes (PMNs) contain lactoferrin (LF), a stable iron binding protein. Arao et al studied its usefulness for screening UTI, defined as the presence of a clinical symptom along with ≥10/mm^3^ PMN and ≥1000 CFU/ml bacteria in a urine sample.^31^ Patients with lower UTI, acute pyelonephritis, and complicated UTI (defined as having underlying urinary tract disease) were included, and urinary LF was measured in patients with UTI and without UTI using an immunochromatographic assay. Subjects with UTI (n = 60) had a high mean urinary lactoferrin at 3.3 μg/ml (95% CI, 2.02–4.58) of urine while in the group without UTI (n = 121) it was 0.030 μg/ml (95% CI, 0.025–0.035) of urine. A cutoff value of 200 ng/ml yielded a sensitivity of 93.3% and specificity of 89.3% for PMNs in the urine.

Secretory immunoglobulin A is produced by plasma cells in mucous membranes. It is also found in the urine of healthy individuals. Deo et al showed in subjects with UTI, there was more than a two standard deviation increase compared with uninfected subjects.^32^ This marker was also increased in subjects with glomerular and anatomical pathology. These data suggest that sIgA may be used as a screening test or in conjunction with other markers for diagnosing UTI.

Other biomarkers such as soluble triggering receptor expressed on myeloid cells-1, urinary myeloperoxidase, α-2 macroglobulin, and the ratio of urinary α-1 microglobulin and serum prostatic antigen have also been studied to diagnose UTI, differentiate between upper and lower UTI and UTI from renal graft rejection.^33^–^36^

Since most of studies done in this group were of poor quality, it is challenging to advocate the use of these markers in clinical practice.

## Discussion

We have conducted an extensive literature review for novel biomarkers that would help in the diagnosis of UTI and assist in establishing its severity. We have identified procalcitonin (PCT) as a potential biomarker that can help in differentiating between lower UTI and pyelonephritis in the pediatric age group.

### Lower UTI vs. pyelonephritis

Procalcitonin (PCT), interleukin 6 (IL-6), and interleukin 8 (IL-8) have been studied to diagnose acute pyelonephritis at an early stage. PCT has shown promising results in this setting. The sensitivity and specificity of PCT ranges from 58% to 94.1% and 36.4% to 93.6%, respectively. These numbers are based on the studies graded as good and fair quality in this review. Most of these studies enrolled children who were less than 1 year to 12 years of age. PCT has not been studied in other populations in the setting of UTI. Serum IL-6 has shown some promise in differentiating between acute pyelonephritis and lower UTI in two studies.^19^,^26^ Both these studies were done in children less than 12 years of age. Interleukin 8 has been studied with negative results in a similar setting.

Approximately 60% of children with febrile UTI develop acute pyelonephritis and 30% of those develop renal scars. 99mTc-dimercaptosuccinic acid (DMSA) scintigraphy is the gold standard used to diagnose renal scars. This is often performed six months after the acute presentation. DMSA is not available in all settings and involves radiation exposure. PCT is a potential biomarker that can be used in settings where DMSA may not be readily available. This can predict renal parenchymal damage at the time of presentation in the pediatric age group. A recent prospective study comparing PCT, ESR and CRP showed superior results with PCT to assess renal parenchymal damage.^37^ Furthermore, a high PCT (≥0.5 ng/ml) has shown to be associated with vesicoureteral reflux in children.^38^ It is a non-invasive test that can help physicians make decisions regarding management and further assessment.^1^ Given the wide ranges in sensitivity and specificity, further study of PCT to distinguish lower UTI from pyelonephritis is warranted.

### Diagnosis of lower UTI

We identified three studies using urine interleukin 8 (IL-8) and lactoferrin (LF) to diagnose UTI. All three studies were fair quality. Urinary IL-8 has been studied in individuals above 18 years of age to diagnose UTI in the post operative setting. This biomarker was also found to be significantly higher in children, less than 12 years of age, with febrile UTI compared with children with fevers because of another cause. Urinary LF has also been studied with positive results to diagnose UTI in adults. To date, leukocyte esterase and nitrite are the two urinary biomarkers investigated most thoroughly. When leukocyte esterase (LE) and nitrite are use together, the sensitivity is 100% (95% confidence interval [CI], 91%–100%), the specificity is 20% (95% CI, 11%–32%), the positive predictive value is 45% (95% CI, 34%–56%), and the negative predictive value is 100% (95% CI, 74%–100%).^15^ Therefore, presence of either or both LE and nitrite does not confirm the diagnosis of UTI. Since the number of studies addressing this problem are few, one cannot advocate the use of urinary IL-8 and LF biomarkers in clinical practice. At this time, however further studies combining the use of LE and nitrite with urinary LF and IL-8 are likely to be helpful.

### Special populations

We were unable to identify studies targeted to populations like individuals with spinal cord injury, diabetics, individuals with polycystic kidney disease, and the elderly (adults 65 years or older). One study specially focusing on kidney transplant recipients was identified.^36^ This study aimed at differentiating between UTI and allograft rejection based on a panel of biomarkers. At this time, no recommendations can be made for diagnosing UTI or ascertaining the severity of infection in these populations.

### Proposed research agenda

To further our ability to diagnose the manifestations of bacterial infections in the urinary tract, the following areas may serve as topics for future research.

- PCT is a promising biomarker in the pediatric age group for diagnosing acute pyelonephritis. This should be studied in other populations as well.
- Serum IL-6 needs to be studied in a larger population to establish its potential as a biomarker to diagnose UTI.
- Target studies to other subgroups including individuals with spinal cord injury, diabetics, individuals with polycystic kidney disease, and the elderly (adults 65 years or older) such that they may have direct applicability to patient care.
- Continued search for additional novel biomarkers, that are specific to the urinary system, to diagnose UTI.

As studies in this field progress, it is conceivable that one may be able to develop a weighted model that uses the information on the available biomarkers. With this information one can compute a cumulative score to differentiate between ASB and UTI and identify renal parenchymal damage earlier on.

## Figures and Tables

**Table 1:** Scoring system for validity based on the STARD criteria.

Validity criterion	Description	Scoring
Participant recruitment	Was recruitment based on presenting symptoms or not?	Presenting symptoms (1) No presenting symptoms (0)
Participant sampling	Was the study population a convenience sample or consecutive series of participants defined by the inclusion and exclusion criteria, setting and locations where data were collected?	Consecutive series (1) Convenience sample (0)
Data collection	Was data collection planned before the index test and reference standard test (i.e. prospectively or retrospectively)?	Prospectively (1) Retrospectively (0)
Reference standard	Was the rationale for reference standard stated?	Stated (1) Not stated (0)
Materials and methods	Were technical specifications of materials and methods including how and when measurements were taken stated?	Stated (1) Not stated (0)
Index test	Were the definition of and rationale for the units, cut offs of the results of the index tests and reference standards stated?	Stated (1) Not stated (0)
Blinding	Readers of index test and reference standard blinded?	Blinded (1) Not blinded or not stated (0)
Completion	Was the number of participants that did not undergo index tests (#test vs. sample size) stated?	Stated (1) Not stated (0)
Time Interval	Was the time interval from index test to reference standard stated?	Stated (1) Not stated (0)

**Table 2:** Score based on STARD criteria.

Reference	Biomarker	Serum/urine	Score
Procalcitonin (PCT)			
Pecile et al^22^	PCT	Serum	8
Guven et al^23^	PCT	Serum	7
Benador et al^18^	PCT	Serum	7
*Gurgoze et al^19^	PCT, IL-1β, IL-6 and TNF-α	Serum	7
Grevaix et al^20^	PCT	Serum	6
Smolkin et al^21^	PCT	Serum	6
Prat et al^39^	PCT	Serum	5
Tuerlinckx et al^40^	PCT	Serum	4
Interleukins (ILs)			
Sheu et al^26^	IL-6 and IL8	Serum and Urine	7
Olszyna et al^24^	IL-8	Urine	7
Sheu et al^29^	IL-1β	Urine	6
Krzemein et al^27^	IL-6 and IL-8	Urine	6
Otto et al^28^	Chemokines	Serum and Urine	6
Jantausch et al^25^	IL-6 and IL-8	Urine	6
Roilides et al^41^	IL-6	Urine	5
Zaki et al^42^	IL-8	Urine	5
Oregioni et al^43^	IL-8	Urine	3
Rao et al^44^	IL-8	Urine	3
Miscellenous			
Fretzayas et al^30^	E-a_1_-Pi	Urine and plasma	8
Arao et al^31^	Lactoferrin	Urine	6
Deo at al^32^	Secretory IgA	Urine	6
Bakokas et al^33^	E-a_1_-Pi	Urine and plasma	5
Smith et al^35^	NBT reduction	Urine	5
Everaert et al^34^	α-1 microglobulin/serum prostatic antigen	Urine-α-1 microglobulin and Serum prostatic antigen	4
Steinhoff et al^36^	Myeloperoxidase, CRP and α2-macroglobulin	Urine-myeloperoxidase and α-2 macroglobulin; serum CRP	3
Determann et al^45^	Soluble triggering receptor expressed on myeloid cells-1	Serum	NA

* Study evaluating PCT, IL-1β, IL-6 and TNF-α.

**Table 3:** Details of scoring.

Validity criterion	Explanation	Scoring	Procalcitonin (PCT)^a^	Interleukins (ILs)^b^	Miscellaneous^c^
Participant recruitment	Was recruitment based on presenting symptoms or not?	Presenting symptoms (1) No presenting symptoms (0)	All studies recruited based on presenting symptoms except one.^40^	All studies recruited based on presenting symptoms except three.^41^,^43^,^44^	All studies recruited based on presenting symptoms except two.^35^,^36^
Participant sampling	Was the study population a convenience sample or consecutive series of participants defined by the inclusion and exclusion criteria, setting and locations where data were collected?	Consecutive series (1) Convenience sample (0)	All studies used consecutive series	All studies used consecutive series except three.^42^–^44^	All studies used consecutive series except three.^31^,^35^,^36^
Data collection	Was data collection planned before the index test and reference standard test (i.e. prospectively or retrospectively)?	Prospectively (1) Retrospectively (0)	Planned and performed prospectively in all studies	Planned and performed prospectively in all except two studies.^43^,^44^	Planned and performed prospectively in all except one study.^35^
Reference standard	Was the rationale for reference standard stated?	Stated (1) Not stated (0)	Rationale for reference standard mentioned in only five studies.^18^,^19^,^21^–^23^	Rationale for reference standard mentioned in all except one study.^41^	Rationale for reference standard mentioned in all except one study.^33^
Materials and Methods	Were technical specifications of materials and methods including how and when measurements were taken stated?	Stated (1) Not stated (0)	Stated in all studies	Stated in all studies	Stated in all studies except two studies.^34^,^35^
Index test	Were the definition of and rationale for the units, cut offs of the results of the index tests and reference standards stated?	Stated (1) Not stated (0)	Not stated in any of the studies	Not stated in any of the studies	Stated in three studies only.^31^,^32^,^35^
Blinding	Readers of index test and reference standard blinded?	Blinded (1) Not blinded or not stated (0)	Stated that the readers of the index test and reference test were blinded in four studies.^18^,^20^,^22^,^23^	Not stated in any of the studies	Stated that readers of the index and reference test were blinded in only two studies.^30^,^35^
Completion	Was the number of participants that did not undergo index tests (#test vs. sample size) stated?	Stated (1) Not stated (0)	Stated in three studies.^19^,^22^,^23^	Stated in all except four studies.^27^–^29^,^43^	Stated in only three studies.^30^,^33^,^35^
Time Interval	Was the time interval from index test to reference standard stated?	Stated (1) Not stated (0)	Stated in all except one study.^23^	Stated in all except three studies.^25^,^42^,^44^	Stated in all except four studies.^32^–^34^,^36^

a Total of 8 studies identified.

b Total of 11 studies identified.

c Total of 8 studies identified.
